# Characterization of a plant-derived monoclonal antibody targeting extracellular enveloped virions of Monkeypox virus

**DOI:** 10.3389/fpls.2024.1481452

**Published:** 2024-11-01

**Authors:** Jennifer A. Melendez, Haiyan Sun, James Bonner, Qiang Chen

**Affiliations:** ^1^ Biodesign Institute, Arizona State University, Tempe, AZ, United States; ^2^ School of Life Sciences, Arizona State University, Tempe, AZ, United States

**Keywords:** Monkeypox virus (MPXV), extracellular enveloped virion (EV), monoclonal antibody, plant-made biologics, plant-made antibody

## Abstract

In 2022, the global outbreak of monkeypox virus (MPXV) with increased human-to-human transmission triggered urgent public health interventions. Plant-derived monoclonal antibodies (mAbs) are being explored as potential therapeutic strategies due to their diverse mechanisms of antiviral activity. MPXV produces two key infectious particles: the mature virion (MV) and the extracellular enveloped virion (EV), both essential for infection and spread. Effective therapies must target both to halt replication and transmission. Our prior research demonstrated the development of a potent neutralizing mAb against MPXV MV. This study focuses on developing a plant-derived mAb targeting MPXV EV, which is critical for viral dissemination within the host and generally resistant to antibody neutralization. Our findings reveal that the mAb (H2) can be robustly produced in *Nicotiana benthamiana* plants via transient expression. The plant-made H2 mAb effectively targets MPXV EV by binding specifically to the A35 MPXV antigen. Importantly, H2 mAb shows notable neutralizing activity against the infectious MPXV EV particle. This investigation is the first to report the development of a plant-derived anti-EV mAb for MPXV prevention and treatment, as well as the first demonstration of anti-MPXV EV activity by an mAb across any production platform. It highlights the potential of plant-produced mAbs as therapeutics for emerging infectious diseases, including the MPXV outbreak.

## Introduction

Monkeypox virus (MPXV) is a zoonotic double-stranded DNA virus belonging to the *Orthopoxvirus* genus and shares close genetic and structural similarities with other genus members including Vaccinia virus (VACV), Variola virus (VARV, the causative agent of smallpox), and ectromelia virus (ECTV) (Lum et al., 2022). Endemic to central and western Africa for decades, MPXV experienced a global resurgence in 2022 through human-to-human transmission, leading to 109,699 confirmed cases worldwide and prompting an international health emergency declaration by the World Health Organization (WHO, 2024). MPXV exists in two infectious forms that facilitate infection: the mature virion (MV) and the extracellular enveloped virion (EV), each of which is responsible for the inter-host and intra-host dissemination, respectively (Schmidt et al., 2012).

Transmission of MPXV occurs through bodily fluids, respiratory droplets, direct contact with infected skin lesions, or contaminated fomites (Lum et al., 2022). The incubation period ranges from 5 to 21 days, and symptoms can include headache, fever, lymph node swelling, and muscle aches. Within 3 days of infection, a rash develops at the infection site, spreading to other bodily areas and resulting in papules or blisters (Lum et al., 2022). Currently, there are no specific approved treatments for MPXV. Antiviral therapies for other orthopoxviruses can be administered to treat MPXV infection under compassionate use policies (Siegrist and Sassine, 2022), although the clinical outcomes and risk-benefit ratio remain uncertain. Smallpox vaccines can also provide cross-protection against MPXV. However, due to the global eradication of smallpox in 1980, the vaccine is no longer routinely used (Gruber, 2022). Ongoing research and development efforts are focused on MPXV-specific treatments, underscoring the critical need for prophylactics and post-exposure therapeutics to protect immunocompromised individuals and those with severe allergic reactions to orthopoxvirus vaccines.

In the past few decades, monoclonal antibodies (mAbs) have emerged as promising protein-based biologics for combating a wide range of diseases due to their high specificity, versatility, and efficacy (Pantaleo et al., 2022). mAbs can be engineered to precisely target specific pathogens or cells, leading to better efficacy and fewer side effects compared to traditional therapies. Given the demonstrated effectiveness of neutralizing antibodies in protecting animals and humans from orthopoxvirus infections (Belyakov et al., 2003; Edghill-Smith et al., 2005; Sang et al., 2023), mAbs present a promising class of therapeutic candidates for combating the re-emerging MPXV epidemic. Our previous research demonstrated the development of a mAb that has potent neutralizing activity against the MV form of MPXV to curb the inter-host transmission (Esqueda et al., 2023). In contrast, there is still a need to develop more efficacious mAbs against the EV form of MPXV as it is generally more resistant to antibody neutralization due to the extra membrane that EVs possess (Vanderplasschen et al., 1998; Law and Smith, 2001). As a result, developing mAbs that can effectively target and neutralize the EV form of MPXV is crucial for achieving comprehensive protection and treatment of MPXV infection. The major viral antigens displayed by the EV of MPXV are A35 and B6, which are homologous to the VACV EV antigens of A33 and B5, respectively (Manes et al., 2008; Wang et al., 2023; Zhao et al., 2024). A35/A33 is particularly noteworthy for its role in viral dissemination within the host and anti-A33 antibodies have demonstrated protective efficacy in susceptible mice against lethal ECTV and VACV infections. Additionally, anti-A33 antibodies have been shown to reduce ECTV viral load in infected organs (Payne, 1980; Cohen et al., 2011; Tamir et al., 2024). Recently, a mAb (H2) has been isolated from A33-specific memory B cells of a volunteer vaccinated against smallpox over 40 years ago (Gu et al., 2022). The H2 mAb has been shown to bind to the A33 antigen of both VACV and ECTV, inhibit ECTV replication, and protect mice from VACV infection (Gu et al., 2022). However, the neutralizing activity of H2 mAb and other anti-A33/A35 mAbs against the EV form of MPXV has not been extensively characterized.

Currently, more than 100 mAbs are approved for therapeutic use (Mullard, 2021). The predominant platform for producing these biologics is based on mammalian cell culture systems, particularly Chinese hamster ovary (CHO) cells. CHO cells are favored for their ability to produce mAbs with high efficiency and human-like glycosylation patterns, which are crucial for antibody efficacy (Dahodwala and Lee, 2019). However, this production platform has several drawbacks, including high production costs and potential risks of contamination by mammalian pathogens. Furthermore, the glycosylation patterns in proteins produced by CHO cells can be heterogeneous, potentially hindering the development of mAbs with optimal effector functions (Sumit et al., 2019). Plants have emerged as a promising alternative platform for the production of mAbs (Chen, 2022). Utilizing plants as expression systems leverages their inherent capacity for rapid growth and biomass accumulation, which can significantly shorten production time and lower production costs compared to traditional mammalian cell culture methods (Chen and Davis, 2016; Nandi et al., 2016). Plant-based production of biologics also reduces the chance of introducing human pathogens during the manufacturing process (Chen, 2011). Furthermore, plant glycoengineering enables the generation of mAbs with a homogenous glycosylation profile, offering the capacity to modulate antibody Fc effector functions such as antibody-dependent cellular cytotoxicity (ADCC) activity and complement-dependent cytotoxicity (CDC) (Chen, 2016; Eidenberger et al., 2023; Esqueda and Chen, 2023).

In this study, we characterized a mAb H2, which was expressed in a glycoengineered *Nicotiana benthamiana* plant line (Strasser et al., 2008), targeting specifically the EV form of MPXV. The H2 mAb was successfully expressed and purified from *N. benthamiana* plants, achieving robust protein expression levels within 5-6 days post infiltration (DPI). Our findings reveal that plant-produced H2 mAb (p-H2 mAb) exhibits strong and specific binding to both MPXV-infected cells and its target antigen. Importantly, H2 mAb also demonstrated neutralizing activity against the EV form of MPXV. These results collectively suggest that plant-produced H2 mAb may be an effective candidate for eliminating MPVX EV virions, highlighting its potential therapeutic application in combating MPXV infections.

## Materials and methods

### Expression vector design and *Agrobacterium tumefaciens* infiltration

The heavy chain (HC) and light chain (LC) of the H2 mAb (Gu et al., 2022) were synthesized by Azenta Life Sciences (Burlington. MA, USA). For targeting the H2 mAb to the apoplast of leaf cells, the calreticulin signal peptide from *Nicotiana plumbaginifolia* (Giritch et al., 2006) was added to the N-terminus of both the LC and HC, without including any ER retention signals in either construct. Full sequences for the LC and HC can be found in the **Supplementary Data Sheet 1**. The synthesized HC and LC fragments were initially cloned into pBluescript KS^+^ and were then cloned into the one-module plant expression vector pBYR11eK2Md as described previously (Jugler et al., 2022a). Positive clones were verified by colony PCR using primers detailed in the **Supplementary Material** and were electroporated into *Agrobacterium tumefaciens* strain EHA105 (Esqueda and Chen, 2021). The EHA105 strain containing the H2 mAb construct was agroinfiltrated into the leaves of glycoengineered *N. benthamiana* plants using a needleless syringe as previously described (Leuzinger et al., 2013; Chen and Lai, 2014). Plants were grown in 65% humidity, at 25°C, with a 16-hour light and 8-hour dark cycle as described previously (Lai and Chen, 2012; Goulet et al., 2019).

### H2 mAb extraction and purification

Leaves of glycoengineered *N. benthamiana* (~ 200 g per batch) were harvested at 5 DPI and homogenized in a buffer containing 1x phosphate-buffered saline (PBS) at pH 5.2, 10 mg/mL Sodium L-ascorbate, 1 mM ethylenediaminetetraacetic acid (EDTA), and 2 mM phenylmethylsulfonyl fluoride (PMSF) as previously described (Fulton et al., 2015; Jugler et al., 2020). The homogenate was filtered through a cheesecloth. The plant protein extract was centrifuged twice 15,000 x g at 4°C for 30 minutes and the pH of the supernatant was adjusted to 5.2 using 0.5N HCl. The protein extract was then incubated at 4°C overnight to precipitate host proteins. Following incubation, the plant extract was clarified by centrifuging three times at 15,000 x g at 4°C for 30 minutes. The pH of the clarified extract was adjusted to 7.0 using 0.5M NaOH and filtered using a 0.2-micron vacuum filter. The p-H2 mAb from plant extract was then purified using Protein A (MabSelect, Cytiva, Marlborough, MA, USA) affinity chromatography using a protocol provided by the manufacturer as we reported previously (Lai et al., 2010; Jugler et al., 2020). At least five batches of H2 mAb purification were conducted.

### SDS-PAGE and western blot analysis

For SDS-PAGE analysis, purified p-H2 mAb was separated under both reducing and non-reducing conditions using 4-20% acrylamide gels and protein bands were visualized with Coomassie Blue R-250 staining as previously described (Jugler et al., 2022b). The purity of the H2 mAb was determined by imaging and quantifying Coomassie blue-stained protein bands on SDS-PAGE using a densitometer as described previously (Jugler et al., 2020). Western blot analysis was performed as previously described (Jugler et al., 2023). Briefly, total leaf soluble proteins were subjected to SDS-PAGE under reducing condition with 10% (v/v) β-mercaptoethanol and non-reducing condition on 4-12% or 12% acrylamide gels. The proteins were then transferred to PVDF membranes at 90 V for 90 minutes) in 1x transfer buffer (25mM Tris, 192 mM glycine, 10% Methanol, pH8.3). The membranes were blocked with 5% milk in 1X PBS containing 0.1% Tween-20 (PBS-T) and incubated for 1 hour at room temperature with goat anti-human kappa conjugated to horseradish peroxidase (HRP) for detecting the LC (Southern Biotech, Birmingham, AL, USA, 1:3,000 dilution) or goat anti-human gamma-HRP for the HC (Southern Biotech, Birmingham, AL, USA. 1:5,000 dilution). Detection was carried out using Pierce Western Blotting Substrate (Thermo Scientific, Waltham, MA, USA) for 5 minutes. Membranes were washed five times with 1X PBS-T at 5-minute intervals, and images were captured using the ImageQuant imaging system (Cytiva, Marlborough, MA, USA).

### ELISA

*1. P-H2 Binding to MPXV A35 Antigen*

An ELISA was conducted to measure the specific binding of p-H2 mAb to the A35 antigen. The coding sequence of the antigen protein was cloned into the pET28a vector and expressed in *E. coli* strain BL21 as described previously (Fang et al., 2006; Yang et al., 2017). The soluble fraction of A35 was purified using immobilized metal affinity chromatography (IMAC) with Ni²⁺ resin, following the manufacturer’s instructions (Thermo Fisher Scientific, Waltham, MA, USA). Purified A35 (200 µl at 1µg/ml) was then coated onto a 96-well plate and incubated at 4°C overnight. The plate was blocked with 5% milk and followed by generic human IgG (SeraCare Life Sciences Inc., Milford, CT, USA, 50µg/ml). The plate was then incubated with p-H2 mAb (5µg/ml) conjugated to HRP using EZ-Link Plus Activated Peroxidase Kit (Thermo Fisher Scientific, Waltham, MA, USA). Detection of p-H2 mAb was performed using an HRP substrate (TMB, SeraCare Life Sciences Inc., Milford, CT, USA), after which the reaction was stopped with 1M H_2_SO_4_. The plate was then read on a spectrophotometer at 450 nm (Biotek Powerwave, Agilent Technologies., Santa Clara, CA, USA) and results were calculated and graphed using GraphPad Prism 10.2.2 (GraphPad, San Diego, CA, USA). The binding ELISA was conducted six times with technical triplicates.

*2. Time Course of H2 mAb expression in plants*

An ELISA that detects the fully assembled mAb was performed to investigate the temporal expression patten of H2 mAb in plants as described (Lai et al., 2014). Glycoengineered *N. benthamiana* plant leaves (at least 20 leaves per time point) were agroinfiltrated (OD_600_ = 0.2) with the expression vector of H2 mAb. Leaves were harvested at 4-9 DPI and stored at -80°C. Once all samples were collected, the leaves were ground for 5 minutes using a chilled mortar and pestle. Protein extracts from each DPI were isolated, clarified using the same extraction buffer and procedure as described in the “H2 mAb Extraction and Purification” section above. Throughout the isolation process, buffers, containers, and procedures were pre-chilled at 4°C and conducted at 4°C to ensure the stability of the H2 mAb. The extracts were then assayed on a 96-well plate coated with the capture antibody (goat anti-human HC, Southern Biotech, Birmingham, AL, USA, 200 µl at 2µg/ml). After 1 hour of incubation at 37°C, the plate was incubated with the detection antibody (a goat anti-human kappa IgG labeled with HRP, Southern Biotech, Birmingham, AL, USA, 0.25µg/ml) for 1 hour at 37°C. The plate was then developed using an HRP substrate (TMB, SeraCare Life Sciences Inc., Milford, CT, USA) and read on a spectrophotometer at 450 nm (Biotek Powerwave, Agilent Technologies., Santa Clara, CA, USA). For each ELISA, serial dilutions of an isotype IgG with known concentrations were included as standards to generate a standard curve, which we used to calculate the concentration of the H2 mAb in the leaves (μg of mAb per gram of fresh leaf weight, FLW). The H2 concentration was then graphed using GraphPad Prism 10.2.2 (GraphPad, San Diego, CA, USA). The time-course experiments were repeated three times. Proteins isolated from each experiment were assayed using ELISA, which was conducted at least twice with technical replicates.

### Virus propagation and cell culture

MPXV strain WRAIR 7-61 (BEI Resources, NR-58622, Rockville, MD, USA) was propagated by infecting T175 cm² flasks of BSC40 cells (American Type Culture Collection (ATCC) CRL-2761, Manassas, VA, USA) at a multiplicity of infection (MOI) of 0.01 for one hour at 37°C in Eagle’s Minimal Essential Medium (MEM) containing 2% fetal bovine serum (FBS) (ThermoFisher, Waltham, MA, USA). After infection, the cells were overlaid with Dulbecco’s Modified-Minimal Essential Medium (DMEM) (ThermoFisher, Waltham, MA, USA) supplemented with 5% FBS. Upon observing viral cytopathic effect (CPE) in 75-100% of cells (approximately 3-4 days post-infection), the virus was harvested by scraping cells into media, centrifuging at 1,000 x g for 10 minutes, and resuspending the pellet in 10 mM Tris, pH 8.0. Crude viral stocks were prepared by freeze-thaw cycles (-80°C, thawed on ice for 30 minutes, then at 37°C for 10 minutes), removing cellular debris by centrifugation at 1,000 x g for 10 minutes, and collecting and aliquoting the supernatant. These crude viral stocks were titrated by infecting confluent monolayers of BSC40 cells with serial dilutions of the viral stock, staining the cells with 0.1% crystal violet in 20% ethanol (MilliporeSigma, Burlington, MA, USA) three days after infection, and then counting plaques. VACV strain Copenhagen (COP) VC2 (kindly provided by Virogenetics) (Jentarra et al., 2008) was prepared similarly as described for MPXV but cultivated in BHK21 cells (ATCC, CCL-10) in MEM with 5% FBS. Additionally, the virus was purified by centrifugation on a 36% sucrose pad at 18,000 x g for one hour at 4°C. Like MPXV, VACV stocks were titrated using BSC40 cells.

### Immunofluorescence staining

BSC40 cells were seeded into a 96-well plate at 50% confluency, infected in triplicate with different dilutions of MPXV or VACV stocks, then fixed with 4% Paraformaldehyde (ThermoFisher, Waltham, MA, USA) in PBS for 15 minutes at room temperature by 24 or 48 hours post infection, respectively. After fixation, the cells were permeabilized with 0.1% Saponin (MilliporeSigma, Burlington, MA, USA) in PBS and stained with 5 µg/ml p-H2 mAb at 4°C overnight. The next day, cells were washed three times with PBS and stained with Alexa488 goat anti-human IgG Kappa (Southern Biotech, Birmingham, AL, USA) at a 1:500 dilution for 1.5 hours at room temperature. Images were captured using the EVOS Cell Imaging System (Thermo Fisher Scientific, Waltham, MA, USA). Experiments were independently repeated three times for both MPXV and VACV.

### Preparation of extracellular enveloped virions

EVs were isolated by infecting a confluent monolayer of BSC40 cells in a 60 mm dish with the indicated virus (MPXV or VACV) at an MOI of 5. The monolayer was washed twice to remove input IMVs, and the cell media supernatant was collected 18-24 hours post-infection. The supernatant was centrifuged twice at 1,000 x g for 10 minutes to remove infected cells. This approach is comparable to, if not more stringent than, previously published protocols for EV isolation (Benhnia Mohammed et al., 2009b). EV stocks were used immediately or stored overnight at 4°C prior to subsequent neutralization assays.

### MPXV and VACV neutralization assay

MPXV EVs were neutralized by diluting fresh MPXV and VACV EV stocks by 10^-3^ and 10^-5^, respectively, and then adding the appropriate volume of antibody or PBS to each dilution. The mixtures of diluted EVs and antibody/PBS were incubated at 37°C for one hour and then applied to confluent monolayers of BSC40 cells in a volume of 0.1mL for an additional hour at 37°C, with rocking every ten minutes. After the one-hour infection, monolayers were washed twice with fresh cell media and then incubated in cell media. MPXV and VACV-infected cells were stained with crystal violet 48 hours (VACV) and 72 hours (MPXV) post-infection. Percent (%) plaque reduction was calculated as: [(number of plaque per well without mAb) − (number of plaque per well with diluted mAb)/(number of plaque per well without mAb) × 100].

### Statistical analyses

Statistical analyses were performed using GraphPad Prism software version 10.2.2. First, the data distribution was assessed using the D’Agostino & Pearson normality test, confirming a Gaussian distribution for all neutralization data. Subsequently, T-tests were employed to compare the neutralization activity of the p-H2 mAb against MPXV and VACV with data from at least two independent experiments with technical triplicates. A p-value of less than 0.05 was considered statistically significant.

## Results

### Expression of H2 mAb in *Nicotiana benthamiana*

**Figure 1:**
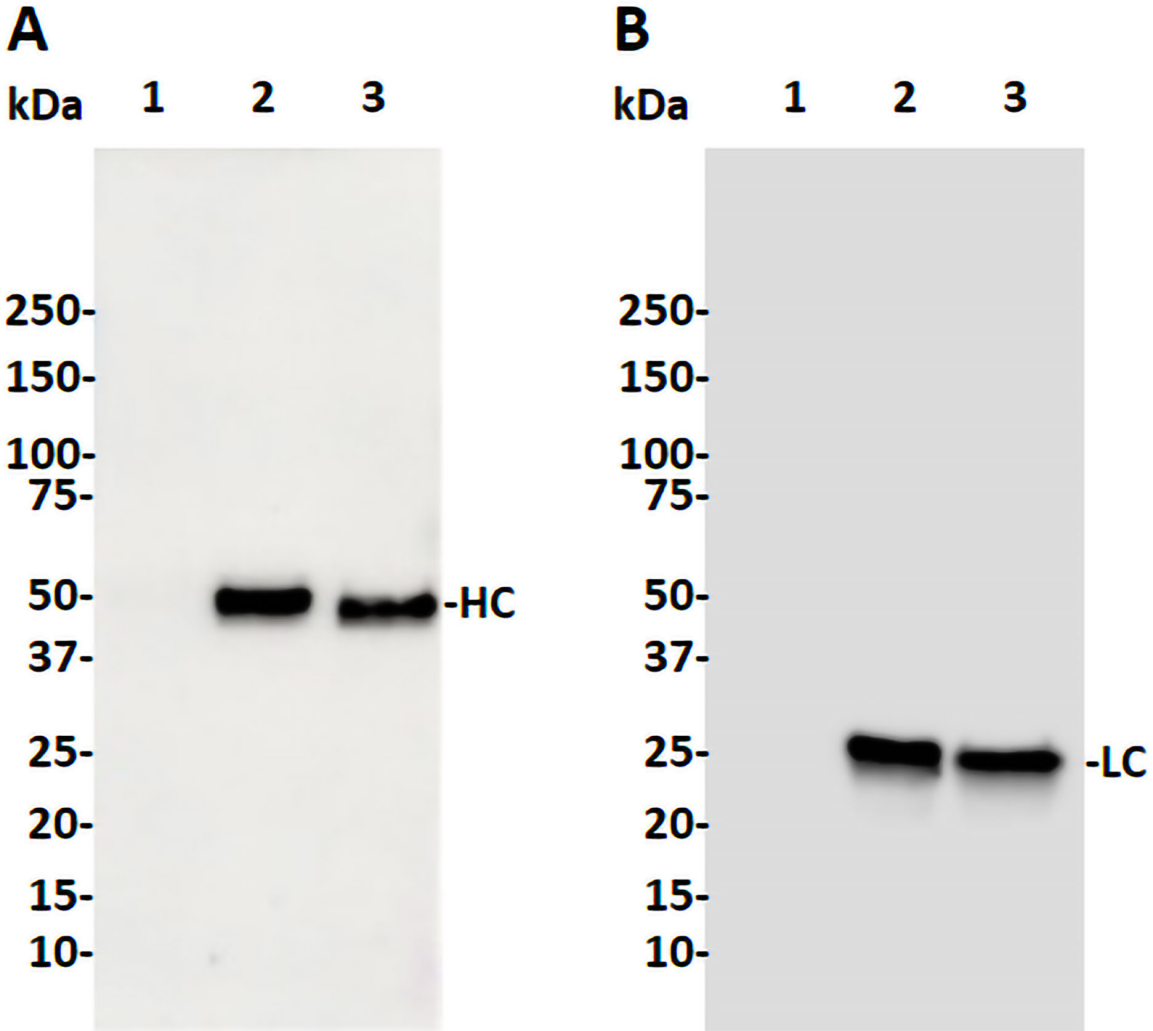
Western blot analysis of the H2 mAb produced in glycoengineered *N. benthamiana* plant. Total proteins were extracted from *N. benthamiana* leaves infiltrated with either the H2 mAb construct or buffer. Proteins were then separated by SDS-PAGE under reducing condition and transferred to PVDF membranes. Immunodetection was performed under reducing condition using antibodies against human gamma HC **(A)** or human kappa LC **(B)**. Lane 1: 20 µg of total proteins from buffer-infiltrated leaves, serving as a negative control. Lane 2: 20 ng of isotype IgG, serving as both a positive control and a loading control. Lane 3: 20 µg of proteins from leaves infiltrated with the H2 mAb construct. One representative blot from multiple experiments is shown.

**Figure 2:**
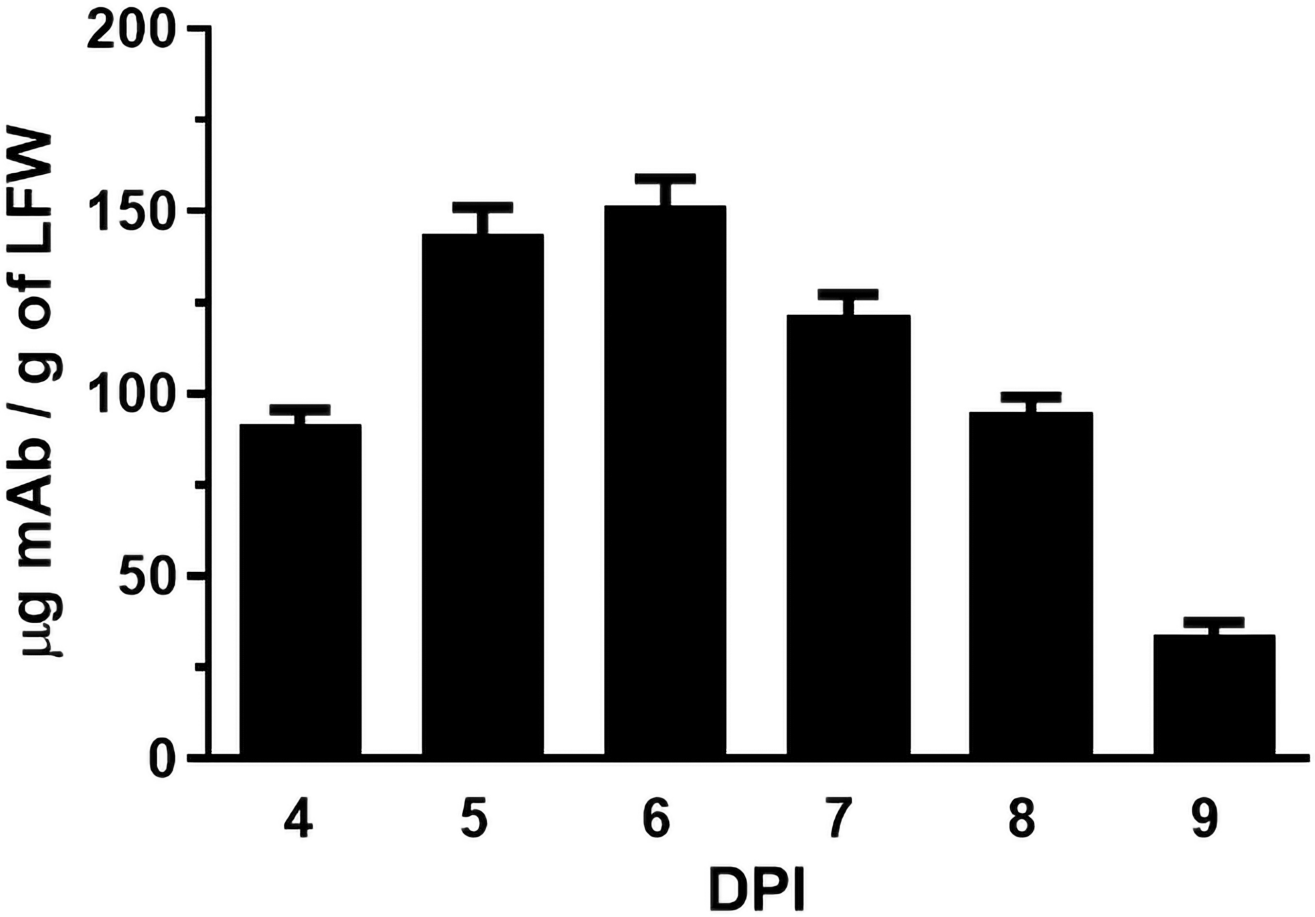
Time course of H2 mAb expression in *N. benthamiana*. Total protein was extracted from glycoengineered *N. benthamiana* leaves agroinfiltrated with the H2 mAb construct at 4, 5, 6, 7, 8, and 9 days post-infiltration. The Levels of H2 mAb were quantified by an ELISA that specifically detects assembled IgG. The data presented are derived from three independent infiltration experiments, with each ELISA repeated twice with technical replicates.

The coding sequences of H2 mAb HC and LC were cloned into the plant expression vector and agroinfiltrated into leaves of a glycoengineered line of *N. benthamiana* plants that forgo the attachment of plant-specific xylose and fucose on their complex N-glycans (Strasser et al., 2008). Western blot analysis validated the expression of H2 mAb in plants, showing detection of the HC and LC at the expected molecular weights of 50kDa and 25 kDa respectively (**Figure 1**). Results obtained under non-reducing conditions indicated that p-H2 assembled into its tetrameric IgG format (**Supplementary Figure S1**), although there were minor, not-fully-assembled fragments that shared a similar banding pattern with the pharmaceutical-grade IgG isotype control (**Supplementary Figure S1**). Furthermore, the temporal expression pattern of p-H2 mAb was investigated by an ELISA that detects only the assembled form of IgG. Our results indicate that the fully assembled mAb accumulated quickly in plants with its peak expression (~ 150 μg of mAb per gram FLW) occurred at 5-6 DPI (**Figure 2**). These results indicate the robust production and assembly of p-H2 mAb in plants.

### Assembly and purification of plant-produced H2 mAb

**Figure 3:**
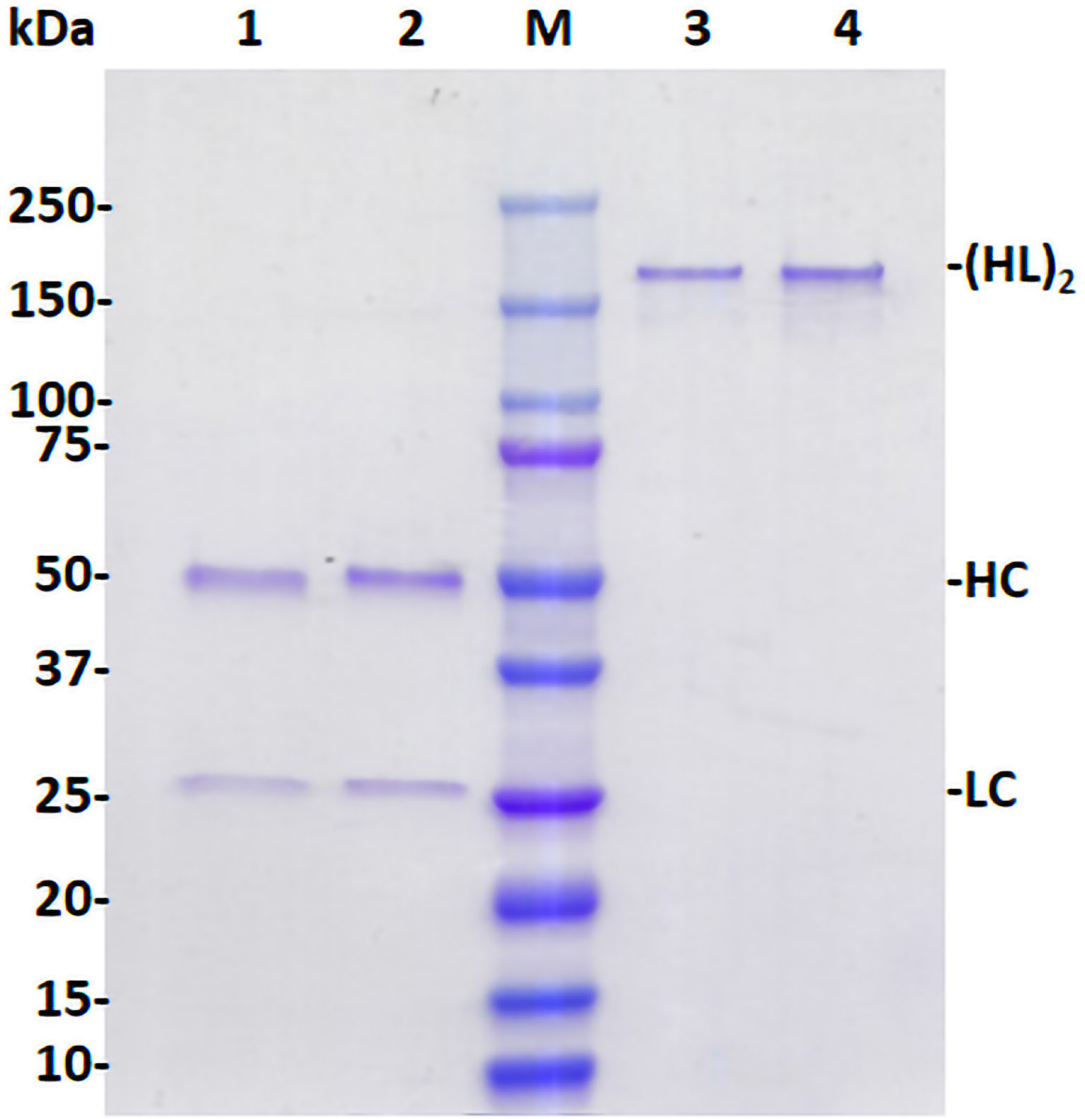
Assembly and purification of plant-produced H2 mAb. The p-H2 mAb was extracted from leaves of glycoengineered *N. benthamiana* plants and purified using Protein A affinity chromatography. Purified p-H2 mAb was analyzed by SDS-PAGE under both reducing (Lanes 1 and 2) and non-reducing conditions (Lanes 3 and 4), and subsequently stained with Coomassie blue. Lanes 1 (3 µg protein) and 3 (1 µg protein) show the plant-produced H2 mAb, while Lanes 2 (3 µg protein) and 4 (1 µg protein) show the Isotype IgG reference produced in CHO cells, serving as both a positive control and a loading control. A single representative result from multiple experiments is presented.

p-H2 mAb was purified by a two-step purification protocol previously developed in our laboratory. SDS-PAGE analysis of purified p-H2 mAb demonstrated that the mAb can be purified to greater than 90% homogeneity by this purification method, which is comparable to that of the control mAb produced in CHO cells (**Figure 3**). No significant degradation products were detected for either LC or HC (**Figure 3**, Lane 1), indicating the integrity of p-H2 mAb. Furthermore, p-H2 mAb was detected as a major band of approximately 170 kDa (**Figure 3**, Lane 3), confirming the assembly of the mAb as indicated by the western blot and ELISA results.

### p-H2 mAb specifically binds to the target Monkeypox virus antigen

**Figure 4:**
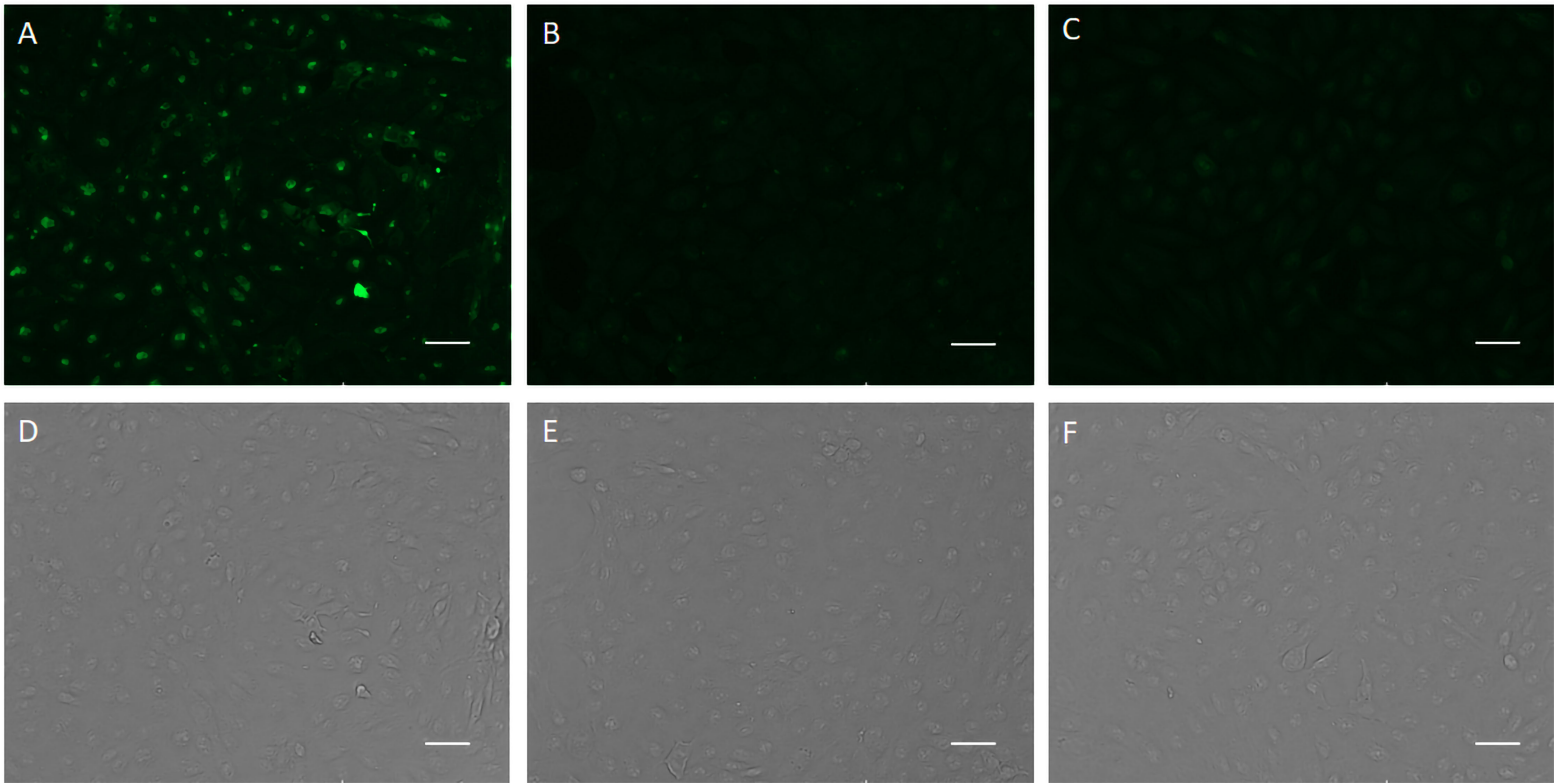
Recognition of viral antigen in MPXV-infected BSC40 cells by p-H2 mAb using immunofluorescence microscopy. MPXV-infected **(A, D)** or uninfected [**(B, E)**, negative control] BSC40 cells in a 96-well plate were fixed and permeabilized, then incubated with p-H2 mAb and subsequently stained with Alexa488-conjugated goat anti-human IgG Kappa. Panels **(C, F)** show MPXV-infected BSC40 cells stained directly with Alexa488-conjugated goat anti-human IgG Kappa, serving as a secondary-antibody-only negative control. Images were captured using an EVOS Cell Imaging System with the EVOS AlexaFluor 488 light cube filter **(A–C)** or under white light settings **(D–F)**. Scale bar represents 50 µm.

**Figure 5:**
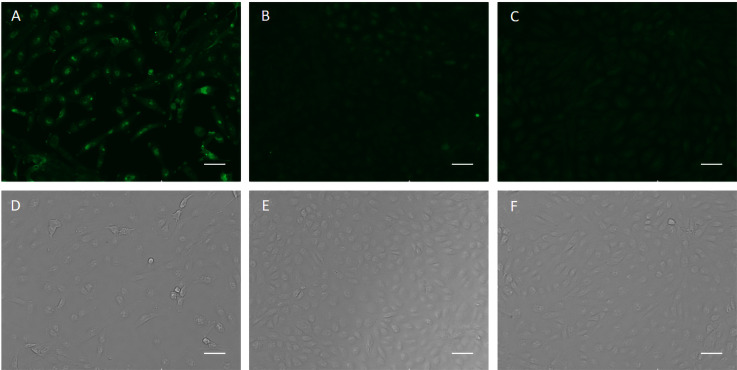
p-H2 mAb recognizes viral antigen in VACV-infected BSC40 cells. BSC40 cells infected with VACV were stained sequentially with p-H2 mAb and the secondary antibody Alexa488-conjugated goat anti-human IgG Kappa **(A, D)** or with the secondary antibody only [**(C, F)**, negative control]. Uninfected BSC40 cells stained with p-H2 mAb and Alexa488-conjugated goat anti-human IgG Kappa **(B, E)** served as an additional negative control. Immunostained images are shown in panels **(A–C)**, with corresponding bright field images in panels **(D–F)**.

**Figure 6:**
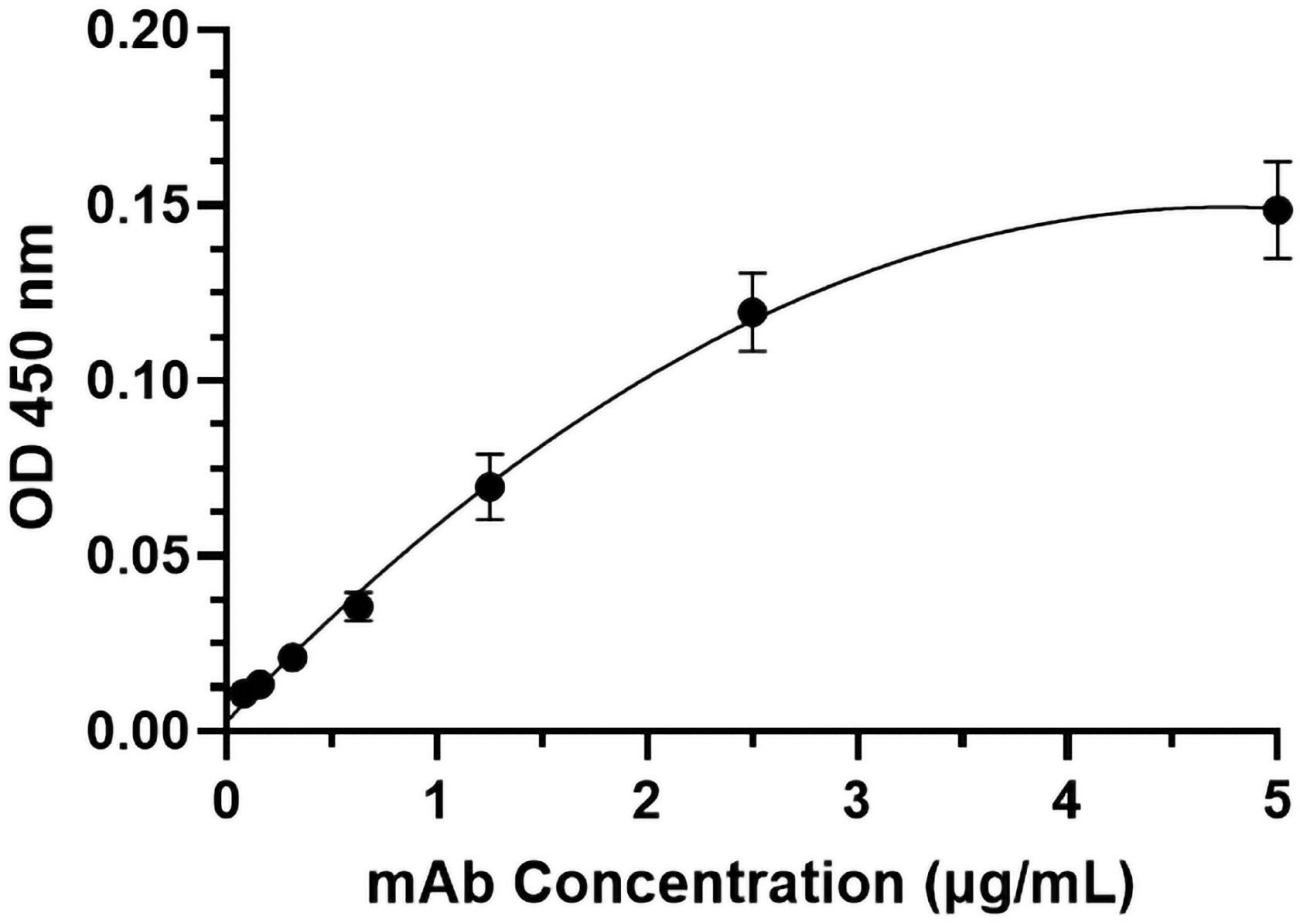
Specific binding of p-H2 mAb to the MPXV A35 antigen. MPXV A35 antigen was immobilized on ELISA plates. After blocking with 5% milk and generic human IgG, the plates were incubated with serial dilutions of HRP-conjugated p-H2 mAb. The mean and standard deviation of absorbance values at 450 nm were measured from six independent experiments, each with technical triplicates, and the data were plotted using GraphPad Prism 10.2.2.

The specific recognition of p-H2 mAb to its cognate antigen was investigated by two independent methods. We first measured its binding to the specific MPXV antigen displayed on the authentic virus. Our results demonstrated that p-H2 mAb exhibits targeted binding to MPXV-infected BSC40 cells (**Figure 4A**), but with no binding observed to uninfected cells (**Figure 4B**) or cells detected with only the secondary antibody (**Figure 4C**). As shown in **Figures 4D–F**, similar cell numbers and distributions were observed under bright-field settings, regardless of whether the cells were infected, uninfected, stained sequentially with primary and secondary antibodies, or stained with only the secondary antibody. Similarly, the p-H2 mAb also demonstrated specific binding to cells infected with the closely related VACV (**Figure 5**). To confirm that p-H2 mAb specifically recognized its target antigen (A35) displayed on MPXV EV, an ELISA was performed with purified A35. As shown in **Figure 6**, p-H2 mAb indeed showed specific binding to A35 antigen. These results demonstrated that p-H2 mAb displayed Fab domains with authentic conformation that can recognize its target antigen in purified form as well as when displayed on the surface of the virion.

### p-H2 mAb neutralizes extracellular enveloped Monkeypox virion

**Figure 7:**
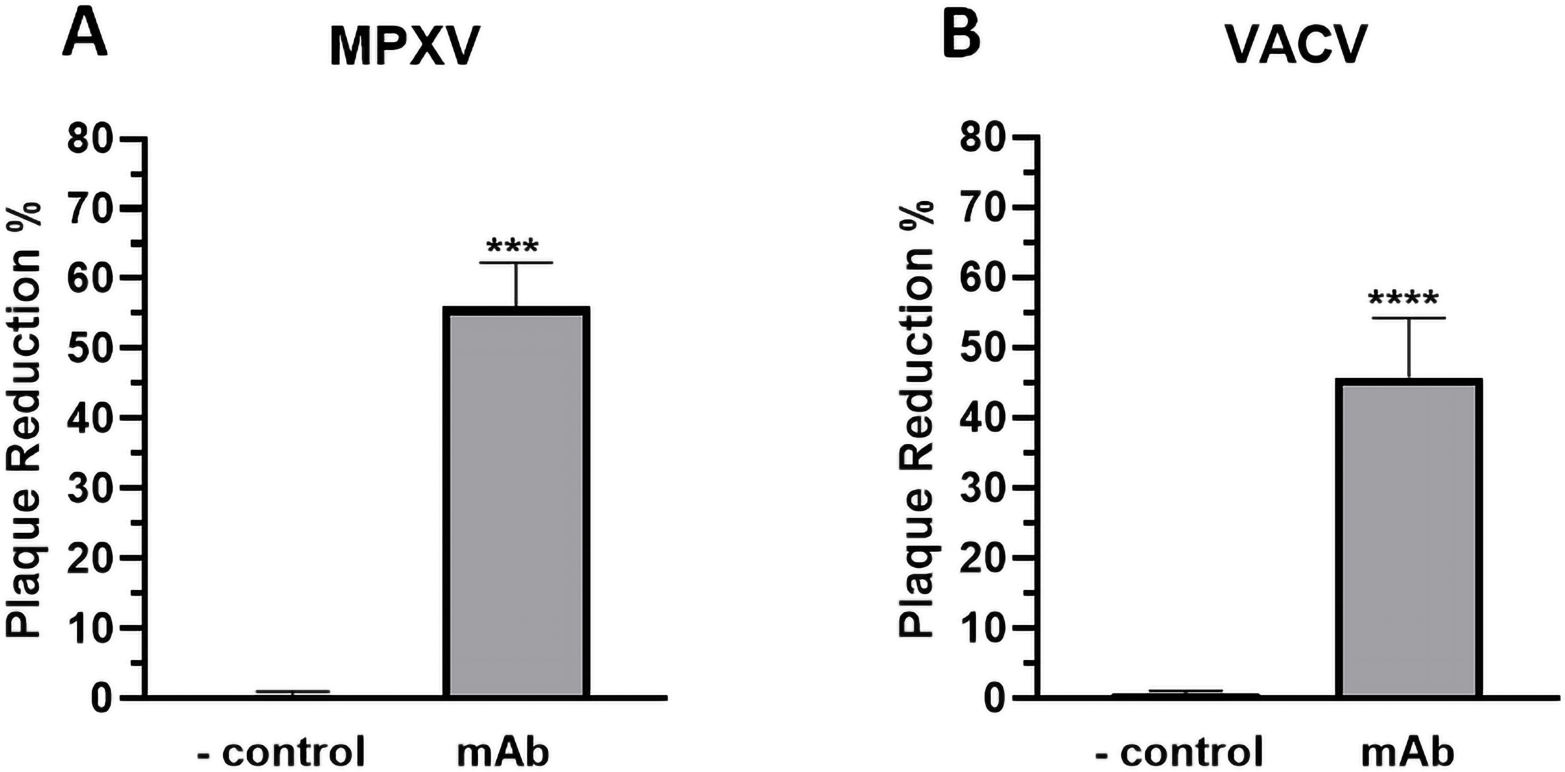
Neutralization of extracellular virion of MPXV and VACV by p-H2 mAb. EVs of MPXV **(A)** or VACV **(B)** were incubated with either PBS (negative control) or p-H2 mAb (25 µg/ml) before being added to BSC40 cells. MPXV- or VACV-infected cells were stained with crystal violet three days or two days later, respectively. Plaque numbers were counted, and plaque reduction percentage (%) was calculated. The results represent data from at least two independent experiments with technical triplicates. *** and **** indicate p values of 0.0001 and < 0.0001, respectively, from T test analysis.

The neutralization potential of p-H2 mAb against the EV virion of MPXV was investigated by a plaque assay as described previously (Denzler et al., 2011; Arndt et al., 2016). As shown in **Figure 7A**, treatment with p-H2 mAb resulted in significant neutralization of MPXV compared to the negative control achieving a 56% plaque reduction (*p* = 0.0001 compared to the negative control). Additionally, the p-H2 mAb also exhibited significant neutralizing activity against VACV, which is similar to that of MPXV (p = 0.14 compared to MPXV), resulting in a 45% plaque reduction when treated with p-H2 mAb (p < 0.0001 compared to the negative control) (**Figure 7B**). These results indicate that p-H2 mAb are functional *in vitro* against the EV form of MPXV as well as that of VACV.

## Discussion

The global MPXV outbreak in 2022 was the largest and most widespread in history, with a significant increase in human-to-human transmission highlighting the urgent need for prophylactics and therapeutics (Lum et al., 2022). These measures are critical to prevent further virus spread and to protect and treat individuals who are allergic to existing MPXV vaccines or unable to mount a protective response to vaccination, especially given the current lack of an FDA-approved therapy specifically for MPXV. mAbs are promising candidates for treating MPXV infection, as their protective efficacy against related orthopoxviruses has been demonstrated in animal models, although research on MPXV remains limited (Belyakov et al., 2003; Edghill-Smith et al., 2005). MPXV produces two distinct types of infectious particles: the MV and the EV. MV, characterized by a single lipid membrane, is predominantly released from infected cells upon cell lysis (Roberts and Smith, 2008; Lum et al., 2022). In contrast, EV buds off from infected cells, acquiring an additional outer lipid envelope that confers resistance to the host’s immune responses, facilitates more efficient viral spread within the host, and helps the virus evade immune detection (Payne, 1980; Schmidt et al., 2012). Consequently, MVs primarily establish initial infection and facilitate host-to-host transmission, while EVs are crucial for viral spread and immune evasion within the host (Schmidt et al., 2012). Accordingly, a successful mAb-based therapy for MPXV should target both MVs and EVs to ensure effective virus neutralization during early infection stages and control viral spread within the host. Failure to target both forms may result in incomplete viral containment, leading to ongoing viral replication and transmission.

Previously, we developed a mAb with potent neutralizing activity against the MV form of MPXV, aimed at preventing inter-host transmission (Esqueda et al., 2023). In this study, we sought to characterize an EV-specific mAb to inhibit viral spreading within the infected host, focusing on the EV-specific antigen A35 from MPXV. The A35 antigen, a homolog of VACV A33, is an envelope glycoprotein, contributing to the formation of action-containing microvilli and facilitating the effective viral dissemination within the host (Perdiguero and Blasco, 2006; Wang et al., 2023). We specifically selected the H2 mAb for further evaluation as a candidate against MPXV EV due to its demonstrated ability to inhibit VACV infection and its recognition of a conserved epitope on both VACV A33 and MPXV A35 (Gu et al., 2022; Yefet et al., 2023). Here, we demonstrated that H2 mAb was rapidly expressed in glycoengineered *N. benthamiana* plants, reaching an accumulation level of approximately 150 μg/gram FLW within 5-6 days post-gene delivery. This expression level is comparable to that of other plant-expressed mAbs driven by the same expression vector (Jugler et al., 2021; Sun et al., 2023). However, further yield enhancement of p-H2 mAb in plants can be achieved through several strategies, such as employing improved expression vectors (Klimyuk et al., 2014) or co-expressing H2 mAb with chaperones (Margolin et al., 2020). Our results also confirm that p-H2 mAb correctly assembled into the expected IgG structure and was purified to a high degree of homogeneity, with no detectable degradation or truncation occurring during its accumulation or purification from plants. We validated the specific binding of p-H2 mAb using two distinct assays. First, we assessed its binding to the MPXV antigen on the authentic virus and found that p-H2 mAb specifically bound to MPXV-infected cells. Similarly, it bound to cells infected with VACV. To further confirm that p-H2 mAb recognizes its target antigen on MPXV EV, we conducted an ELISA using purified A35 EV antigen. The results confirmed that p-H2 mAb specifically binds to the A35 antigen, indicating proper folding and recognition of its target antigen in both purified form and on the surface of the virion. Importantly, p-H2 mAb exhibits neutralization activity against both live MPXV and VACV, with comparable potency to each other and to that reported for ECTV (Gu et al., 2022). Given that the mammalian cell-produced H2 mAb has shown significant protective efficacy against lethal VACV infection in mice (Gu et al., 2022), the similar neutralizing potency against MPXV suggests that p-H2 mAb may also be effective *in vivo* as a prophylactic or therapeutic agent for preventing or treating MPXV infection.

Neutralizing MPXV EV with antibodies is challenging primarily due to the EV’s additional outer lipid envelope. Studies with VACV have shown that direct antibody neutralization of EVs is inefficient even at high antibody concentrations (Vanderplasschen et al., 1998; Galmiche et al., 1999; Law and Smith, 2001; Bell et al., 2004; Viner and Isaacs, 2005). However, further studies reveal that anti-EV antibodies can provide protection through mechanisms beyond neutralization, such as Fc effector functions. For example, polyclonal antibodies against A33 and B5 have been shown to engage the complement system, eliminating VACV EVs via CDC activity through opsonization and virolysis (Lustig et al., 2004; Cohen et al., 2011). Additionally, Fc receptor engagement and ADCC activity of anti-EV antibodies have been documented (Benhnia Mohammed et al., 2009a, Benhnia Mohammed et al., 2009b; Cohen et al., 2011). Given that Fc effector functions depend on the N-glycosylation of the antibody’s Fc domain (Kellner et al., 2017; Steffen et al., 2020), this evidence suggests that our plant-based system may provide an opportunity to enhance the efficacy of anti-MPXV EV mAbs. Plant glycoengineering has enabled the production of mAbs with a homogeneous N-glycosylation profile, a level of uniformity that current mammalian-cell-based systems cannot achieve (Chen, 2016). Since p-H2 mAb was produced in the glycoengineered GnGn plant line, it is expected to exhibit the same uniform GnGn N-glycosylation structure as other mAbs produced in this line of *N. benthamiana* (Jugler et al., 2022a, Jugler et al., 2023; Sun et al., 2023). Afucosylated GnGn glycoforms have been shown to enhance CDC and ADCC activity via increased binding to Fc-gamma receptors (Sun et al., 2019; Yu et al., 2021; Yang et al., 2023). Thus, p-H2 mAb may offer additional mechanisms of MPXV elimination *in vivo* in addition to neutralization, supporting the hypothesis that p-H2 mAb might have better *in vivo* efficacy compared to mammalian cell-produced H2 mAb due to enhanced Fc effector functions. While plant host engineering has produced plant lines with several advantages over mammalian systems for producing mAbs with defined human N-glycans, challenges remain. One such challenge is underglycosylation, which is occasionally observed in plant-produced IgGs (Chen, 2016). This issue can be addressed by co-expressing oligosaccharyltransferases—enzymes that transfer preassembled oligosaccharides to polypeptides in the endoplasmic reticulum (Chen, 2016). Additionally, as a relatively new production platform, there has been uncertainty regarding regulatory hurdles in approving glycoprotein biologics made in plants. Fortunately, results from human clinical trials of plant-made glycoproteins have shown that they are not particularly immunogenic, and even the presence of plant-specific glycans does not induce any unwanted side effects (Shaaltiel and Tekoah, 2016; Rup et al., 2017).

This study is the first to report the development of an anti-EV mAb in plants for preventing and treating MPXV infection, as well as the first demonstration of anti-MPXV EV activity by an mAb across any production platform. In the next phase of the study, we plan to perform comparative analyses of the CDC and ADCC activities of various H2 mAb glycovariants, including those produced in mammalian cells, and assess their efficacy in animal models. These studies will provide valuable insights into the full potential of plant-produced H2 mAb. It would also be worthwhile to investigate potential synergistic effects with a therapeutic cocktail comprising p-H2 anti-EV mAb and anti-MV mAb against MPXV. These analyses could provide valuable insights into how different viral proteins dictate the requirements for the host to eliminate the virus using various mechanisms and how Fc effector functions contribute to protection *in vivo*. As H2 mAb was isolated from a volunteer vaccinated with the VARV vaccine and has demonstrated inhibitory activity against both VACV and ECTV, our findings on its activity against MPXV further expand its effectiveness against another orthopoxvirus, supporting its potential as a broad-spectrum, pan-orthopoxvirus therapeutic. In August of 2024, the WHO re-declared that the MPXV epidemic a Public Health Emergency of International Concern. As MPXV outbreaks continue, up to 90% of cases occur in people with human immunodeficiency virus (HIV) (Saldana et al., 2023), who experience in more severe symptoms than those without HIV (Chastain et al., 2023). Therefore, it is crucial to monitor emerging mutants and quickly develop effective therapeutics against them. We propose that the plant-based system may be one of the best platforms to rapidly develop therapeutic mAbs that maintain efficacy against potential mutants via Fc effector functions and quickly screen for mAb cocktails with synergistic interactions. In summary, this study demonstrates the potential of using anti-EV mAbs as therapeutics against MPXV infection and highlights the utility of plant biotechnology in developing biologics against viral infections.

## Data Availability

The original contributions presented in the study are included in the article/**Supplementary Material**. Further inquiries can be directed to the corresponding author/s.
