# Supplementary material for: Characterization of a plant-derived monoclonal antibody targeting extracellular enveloped virions of Monkeypox virus
Source: Front Plant Sci. 2024 Nov 1;15:1481452. doi: 10.3389/fpls.2024.1481452 (PMC11563991; doi:10.3389/fpls.2024.1481452)
Supplement: Supplementary file 1 [file DataSheet1.pdf]

Supplementary Data Sheet 1

Heavy chain sequence of H2

EVQLVQSGGGLVQPGGSLRLSCAASGITFSTYAMSWVRQAPGKGLEWVSAIGGSGSRTYYGDSVKGRFTI
SRDNSKNTLYLQMNSLRAEDTAIYYCAKVFRDSSGYYGGFDDWGQGTLLTVSSASTKGPSVFPLAPSSKST
SGGTAALGCLVKDYFPEPVTVSWNSGALTSGVHTFPAVLQSSGLYSLSSVVTVPSSSLGTQTYICNVNHKPS
NTKVDKKVEPKSCDKTHTCPPCPAPELLGGPSVFLFPPKPKDTLMISRTPEVTCVVVDVSHEDPEVKFNWY
VDGVEVHNAKTKPREEQYNSTYRVVSVLTVLHQDWLNGKEYKCKVSNKALPAPIEKTISKAKGQPREPQVY
TLPPSRDELTKNQVSLTCLVKGFYPSDIAVEWESNGQPENNYKTTPPVLDSDGSFFLYSKLTVDKSRWQQG
NVFSCSVMHEALHNHYTQKSLSLSPGK

Light chain sequence of H2

DIQLTQSPSSLSASVGDRVTITCRPSQGVSRWLAWYQQKPGKAPKFLIYAASSLQSGVPSRFSGSGSGTDF
TLTINSLQPEDFATYYCQQANSFPWTFGQGTKVEIKRTVAAPSVFIFPPSDEQLKSGTASVVCLLNNFYPREA
KVQWKVDNALQSGNSQESVTEQDSKDSTYSLSSTLTLSKADYEKHKVYACEVTHQGLSSPVTKSFNRGEC

5’ primer for verifying the positive clone of H2 mAb

ACTCGAGAAACAAACAAAATCAACAAATATAGAAAATAACG

3’ primer for verifying the positive clone of H2 mAb

CTTCTTCTTCTTCTTTTCTCATTGTC

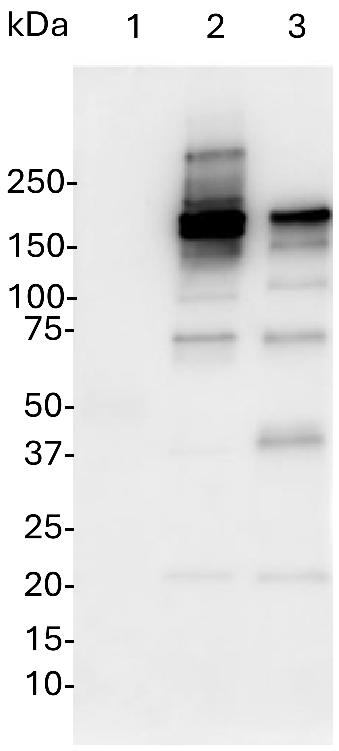

Figure S1. Western blot analysis of the H2 mAb produced in glycoengineered *N. benthamiana* plant. Total proteins were extracted from plant leaves infiltrated with either the H2 mAb construct or buffer. Proteins were then separated by SDS-PAGE under non-reducing condition and transferred to PVDF membranes. Immunodetection was performed under reducing condition using antibodies against human kappa LC . Lane 1: total proteins from buffer-infiltrated leaves, serving as a negative control. Lane 2: isotype IgG, serving as both a positive control. Lane 3: proteins from leaves infiltrated with the H2 mAb construct. One representative blot from multiple experiments is shown.
